# HIV-1 Drug Resistance, Distribution of Subtypes, and Drug Resistance-Associated Mutations in Virologic Failure Individuals in Chengdu, Southwest China, 2014-2016

**DOI:** 10.1155/2020/5894124

**Published:** 2020-03-23

**Authors:** Jiayi Chen, Yang Liu, Sijing Liu, Dan Yuan, Ling Su, Li Ye, Fanghong Gong, Yushuang Gao, Saira Baloch, Xiaofang Pei

**Affiliations:** ^1^Research Center for Occupational Respiratory Diseases, West China School of Public Health (No. 4 West China Teaching Hospital), Sichuan University, 16#, Section 3, Renmin Road South, Chengdu, 610041 Sichuan, China; ^2^Chengdu Center for Disease Control and Prevention, 4#, Longxiang Road, Chengdu, 610041 Sichuan, China; ^3^Department of Public Health Laboratory Sciences, West China School of Public Health (No. 4 West China Teaching Hospital), Sichuan University, 16#, Section 3, Renmin Road South, Chengdu, 610041 Sichuan, China; ^4^Sichuan Center for Disease Control and Prevention, 16#, Xiaoxue Road, Chengdu, 610041 Sichuan, China

## Abstract

The National Free Antiretroviral Therapy (ART) Program in China has initiated to provide ART to HIV-1 patients, which has acted as an efficient method to suppress viral replication and helps prevent onward transmissions. But the problems of HIV drug resistance (HIVDR) may also come along. There is little data on the prevalence of HIVDR in Chengdu, where the number of HIV/AIDS patients ranks first among provincial capitals. Therefore, epidemiological surveillance was conducted in this area. From 2014 to 2016, HIV/AIDS patients (15 years and older) who had received first-line ART for at least six months were enrolled. Demographic, behavioral information and medical history were recorded, and blood samples were collected for viral loads and immune cell count analyses. HIV-1 *pol* was obtained for HIV-1 subtypes and drug resistance-associated mutations (DRMs) among virologic failure patients. A total of 13,782 individuals were enrolled, and 481 samples were sequenced for subtypes and drug resistance analysis. Six subtypes were identified, among which CRF01_AE (54.3%) and CRF07_BC (41.6%) were the dominant subtypes, and CRF55_01B (0.4%) was detected in Chengdu for the first time. The prevalence of HIVDR in treatment-experienced patients was 1.8%, with 1.2% to nucleoside reverse transcriptase inhibitors (NRTIs), 1.7% to non-NRTIs (NNRTIs), and 0.14% to protease inhibitors (PIs). The leading DRMs observed in the study were M184I/V (59.59%) against NRTIs and K103N (37.55%) against NNRTIs. This study focused on the HIVDR surveillance among patients receiving treatment in Chengdu. The overall prevalence of HIVDR was relatively low among treated patients. These findings were believed to be contributed to an understanding of HIV-1 subtypes, HIVDR prevalence, and DRMs in Chengdu and thereby optimizing clinical management, prevention, and control of HIV.

## 1. Introduction

AIDS, also known as “acquired immunodeficiency syndrome,” is a highly infectious disease caused by human immunodeficiency virus (HIV). It has been widely spread around the world, with approximately 37.9 million people living with HIV worldwide at the end of 2018 [1]. The situation of the HIV epidemic is a growing burden to public health in China. As of August 2018, it was reported that 841,478 people were living with HIV/AIDS in China, and the number of deaths was 259,200, ranking first among officially reported infectious diseases [2].

Application of highly active antiretroviral therapy (HAART) has significantly reduced the transmission of HIV and has decreased HIV-related morbidity and mortality [3–5]. By the end of 2018, 23.3 million people infected with HIV had been receiving antiretroviral therapy globally [1]. China has launched the National Free ART Program in 2002, which has acted as an efficient way of treatment for HIV. However, as accessibility of ART increases and improved survival rate occurs, it may arouse concerns for HIV drug resistance (HIVDR) [6–9]. HIVDR poses a serious threat to the prevention of transmission of HIV/AIDS due to compromised efficacy of the first-line regimen. In addition, drug-resistant strains can be transmitted to newly infected individuals and are likely selected as dominant strains. HIVDR surveillance has made it possible to detect DR strains in time, evaluate and adjust treatment regimens, and reduce the transmission of DR strains.

Previous investigations around the world have shown that the prevalence of HIVDR varies in different regions, during different time periods and in different target populations. WHO reported that the prevalence of any HIVDR among all individuals receiving treatment ranged from 3% to 29% [10]. In the past few years, the proportion of HIVDR in China has remained relatively low, at 5.4% in 2011 and 1.1% in 2015 among treatment-experienced patients [11]. However, these reports are sporadic and limited, and continuous monitoring is still needed.

Sichuan is a representative province in Southwest China and is adjacent to Yunnan and Tibet Autonomous Region. Chengdu is the capital city of Sichuan Province and acts as the political, economic, and cultural center of this area. National surveillance shows that HIV infection rates are incredibly high in Southwest China and increases in HIV epidemics among men who have sex with men (MSM) have been especially rapid in urban centers [12], especially in Chengdu, where HIV prevalence in this population has remained high and the overall HIV prevalence was 15.5% from 2009 to 2014 [13], twice the national average among MSM (6.3%) [14].

The first HIV-1 case in Chengdu was reported in 1992. By the end of 2016, a total of 18,603 people were living with HIV in Chengdu, which accounts for the largest HIV/AIDS population among other provincial capitals, and 15,633 patients were receiving ART. However, there were little data on the molecular epidemiology of HIV-1 and the prevalence of drug resistance-associated mutations (DRMs) in this area. Therefore, epidemiological surveillance was conducted in patients receiving ART treatment in Chengdu, to provide HIV-1 subtypes, the prevalence of DR, and DRM profiles and thus to optimize clinical management, prevention, and control of HIV.

## 2. Methods

### 2.1. Study Design, Participants, and Specimen

This study was carried out at the Center for Disease Control and Prevention at Chengdu, Sichuan Province, West China. From 2014 to 2016, HIV/AIDS patients (15 years and older) who had received first-line ART for at least 6 months were enrolled in this study. The patients who were on second-line ART (<6 months) were excluded. Blood samples were collected in EDTA containers, and plasma was extracted and cryopreserved for analyses. Patients or their guardians (patients under 18 years old) who provided written informed consent participated in the study. Demographic, behavioral information and medical history were recorded using a standard questionnaire.

### 2.2. Immune Cell Count and HIV-1 Viral Load Assay

To assess immune response, CD4^+^ T cell count was measured by a flow cytometer BD FACSCount™ System (Becton Dickinson, Franklin Lakes, N.J., USA). HIV-1 RNA viral load (VL) was quantified with an Abbott RealTime HIV-1 Amplification Reagent Kit (Abbott Molecular, Chicago, USA) and NUCLISENS EASYQ HIV-1 2.0 (BioMérieux, France) according to the manufacturer's instructions. Virologic failure was defined as measurement of VL above 1000 HIV RNA copies/ml, and then, HIV-1 *pol* gene was analyzed.

### 2.3. Amplification of HIV-1 *pol* Gene

HIV viral RNA was extracted from plasma using the Abbott RealTime HIV-1 Amplification Reagent Kit and then was reverse transcripted into cDNA by using the AccessQuick™ RT-PCR System (Promega, Madison, Wisconsin, USA). The *pol* gene fragments, containing the entire protease gene and partial reverse transcriptase gene (codons 1-300), were amplified by nested PCR (2x Pfu PCR MasterMix, Tiangen, Beijing, China). Primers and cycling conditions were previously described [15]. The DNA fragments were identified by 1.0% agarose gel electrophoresis. Then, the products were purified and sequenced by Sangon Biotech Co., Ltd. (Shanghai, China).

### 2.4. Drug Resistance-Associated Mutation Analysis

The obtained nucleotide sequences were assembled, edited, and aligned using ChromasProl.33 and BioEdit 7.0. HIV-1 subtyping was performed by constructing the HIV-1 *pol* phylogenetic tree (MEGA 5.0). The HIVDB Program (https://hivdb.stanford.edu/hivdb/by-sequences/) from the Stanford HIV Drug Resistance Database was used to analyze the sequences for DRMs [16]. Drug resistance was divided into five levels: sensitive, potentially resistant, low resistant, intermediate resistant, and high resistant.

### 2.5. Statistical Analysis

Statistical analysis was performed using SPSS Statistics version 22.0. Categorical variables were described in numbers and proportions. Possible associations of HIV-1 subtypes, HIV-1 drug resistance with demographic, exposure category, and clinical variables were analyzed by using the Chi-square test or Fisher's exact test and logistic regression. All tests were two-sided with statistical significance at *p* < 0.05.

## 3. Results

### 3.1. Demographic Characteristics of Study Participants and the Prevalence of HIV-1 Subtypes

Between 2014 and 2016, a total of 13,872 HIV-infected patients were tested for HIV-1 VL. 4.7% (653/13872) of cases were considered treatment failure. A total of 481 (481/653) samples were amplified and sequenced successfully for subtypes and genetic resistance. Most of the subjects were male (411/481, 85.4%), and the main route of infection was heterosexual contact (342/481, 71.1%). The median age was 41 years (range: 15-81years), of which 50.3% were married. The demographic and subtype distributions of 481 patients are presented in Table 1. Based on the generated sequences, six subtypes were identified. CRF01_AE (261/481, 54.3%) and CRF07_BC (200/481, 41.6%) were found to be the predominant subtypes. Other subtypes such as CRF08_BC (9/481, 1.9%), B (6/481, 1.2%), C (3/481, 0.6%), and CRF55_01B (2/481, 0.4) were found in small proportions.

### 3.2. Prevalence and Risk Factors of Drug Resistance

According to the HIV Drug Resistance Database, 245 DR cases were identified in 481 sequences. The prevalence of DR from 2014 to 2016 was 1.8% (245/13872) in treatment-experienced patients and 37.5% (245/653) in virologic failure patients. The comparison of characteristics between patients with and without DR is listed in Table 2. Patients with HIVDR were mostly male (85.3%, 209/245), and half of the patients were 25 to 45 years old (50.6%, 124/245), married (52.2%, 128/245), and majority of them contracted HIV via heterosexual route (71.8%, 176/245). Half of the cases have been treated for 1-3 years (52.2%, 128/245), most of whom received TDF+3TC+NVP/EFV regime and were infected with CRF01_AE (68.2%, 167/245).

Univariate analyses were conducted to correlate demographic characteristics with drug resistance (Table 2). No differences were found between the prevalence of DR and gender, age at diagnosis, marriage status, infection routes, treatment duration, treatment change, and VL (all *p* > 0.05). CD4^+^ T cell count in the DR group was statistically lower than in that without the DR group (*p* < 0.00). CRF01_AE and “TDF+3TC+EFV/NVP/other” are associated with DR (*p* < 0.00). A logistic regression analysis showed that there was no factor significantly associated with HIVDR.

### 3.3. Categories of Antiretroviral Drugs and Susceptibility of Drug Resistance

**Figure 1:**
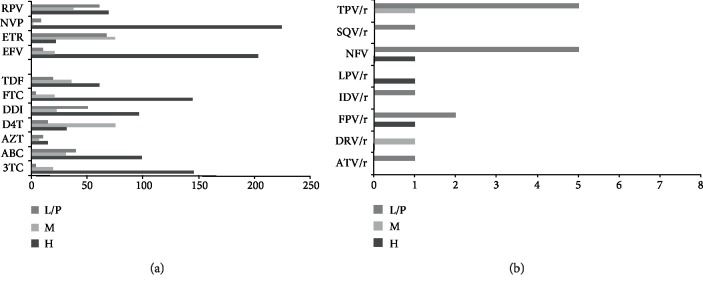
The categories of antiretroviral drugs and their susceptibility of resistance level. Numbers of patients with DR by NRTIs and NNRTIs (a), PIs (b) and their susceptibility of resistance level. H: high resistance, M: moderate resistance, L/P: low or potential resistance.

In total, 1.2% were resistant to nucleoside reverse transcriptase inhibitors (NRTIs), 1.7% to non-NRTIs (NNRTIs), and 0.14% to protease inhibitors (PIs). The NRTI-associated mutations were forecasted to be highly resistant to lamivudine (3TC, 67.8%, 146/245) and abacavir (ABC, 40.4%, 99/245), resistant to stavudine (D4T, 50.2%, 123/245) and tenofovir (TDF, 47.76%, 117/245), and intermediate or low resistant to azidothymidine (AZT, 7.3%, 18/245). 96.33% of the patients were predicted to have high resistance to efavirenz (EFV) and nevirapine (NVP), followed by resistance to rilpivirine (RPV, 67.0%, 169/245) and etravirine (ETR, 67.3%, 165/245) (Figure 1). Twenty cases were identified to have drug resistance to PI drugs, among which 15 cases belong to low or potential resistance.

### 3.4. The Degree of Drug Resistance Profiles

The prevalence of all DRMs to NRTIs, NNRTIs, and PIs was displayed in Supplementary [Supplementary-material supplementary-material-1]. 14 DRMs were observed in NRTIs. The most commonly observed mutations with NRTIs were M184I/V (59.59%, 146/245), K65R (28.16%, 69/245), D67N/G (19.18%, 47/245), K70E/K/R (17.14%, 42/245), Y115F (15.10%, 37/245), L74I/V (11.02%, 27/245), and T215I/Y (8.16%, 20/245). 16 DRMs were found in NNRTIs. K103N (37.55%, 92/245) was the most frequent mutation, followed by G190A/E/K/Q/S/V (28.57%, 70/245), V179I/D/E/T (27.76%, 68/245), V106A/I/M (26.12%, 64/245), Y181C/V (18.78%, 46/245), K101E/H/P (14.69%, 36/245), Y188C/H/L (5.71%, 14/245), L100I (4.08%, 10/245), and M230L (4.08%, 10/245). Three major PI-associated and 7 secondary DRMs were found in this study. The most frequent mutations were L10I/V (32), A71I/T/V (28), and K20I/R (26), among which L10I/V and A71I/T/V are mutations that do not affect drug susceptibility and K20I/R were predicted to have potential resistance to NFV.

### 3.5. The Distribution of DRMs among Different HIV-1 Subtypes

14 NRTI-associated DRMs were all found in CRF01_AE, and V75I/L/M, T69N/D, and L210W were not found in CRF07_BC (S [Supplementary-material supplementary-material-1]). 16 and 15 (except A98G) NNRTI-associated DRMs were found in CRF01_AE and CRF07_BC, respectively. No significant difference was found in the distribution of NRTI/NNRTI-associated DRMs and CRF01_AE and CRF07_BC subtypes. NNRTI-associated DRMs with extensively drug resistance such as G190A/E/K/Q/S/V, V179I/D/E/T, Y181C/V, and K101E/H/P reside in CRF55_01B and subtype B, C. Only one case infected with CRF_08BC recombinant subtype showed DR. E138A/G/R/K/Q was found in this case and was predicted to have low/potential resistance to RPV and ETR. PI-associated mutations only resided in CRF01_AE and CRF07_BC. The distribution of mutations in the PI coding region was significantly different in the two recombinant subtypes (*p* < 0.05). One patient infected with CRF07_BC recombinant subtype showed M46I, I47A, and I50V 3 primary mutations, which was predicted to be resistant to all PIs.

## 4. Discussion

Since the 1980s, pioneers have focused on the issues of HIVDR [17] and technical protocols for HIVDR have been gradually developed with sustained efforts. China has also compiled professional standards such as “Monitoring Strategies and Detection Techniques of HIVDR” to facilitate the comparison and application of various findings. From 2014 to 2016, 1793, 6005, and 6074 of HIV/AIDS patients receiving ART were examined for HIV-1 VL in Chengdu. HIV-1 *pol* genes of 481 samples were sequenced in this study, and thus, HIV-1 subtypes, the prevalence of DR, and profiles of DRMs were investigated.

Chengdu is an area with predominance of CRF01_AE and CRF08_BC, indicating distinct heterogeneity compared to what has been shown in other regions [18, 19]. For instance, about half of the subtypes were identified as CRF08_BC (47.4%) among the recently infected population in Yunnan [20] and CRF07_BC was the predominant strain in Xinjiang Province [21]. However, CRF01_AE was dominant in east China [22, 23]. It has been confirmed that one of the drug-trafficking routes in mainland China is from Yunnan to Sichuan and then to Xinjiang [24]. Hence, it is assumed that subtype profile in Chengdu is similar to Yunnan and Xinjiang provinces.

In addition, novel CRF such as CRF55_01B was first discovered in Chengdu, and both cases were MSM. CRF55_01B, a recombinant form of CRF01_AE and subtype B, was first identified in 2012 among MSM in China, becoming the third CRF in China [25, 26]. The prevalence of this novel CRF ranged from 0.7% in Beijing to 14.6% in Guangzhou [27, 28] and has increased in the past 5 years especially in South and East China [29]. Recently, more unusual CRFs derived from CRF55_01B isolates are being detected, which specified the complexity of HIV-1 strains [30]. Large cities may be the places where new recombinant strains would originate and spread. With CRF55_01B emerging in this area, long-term molecular monitoring is necessary for control and prevention of the HIV-1 epidemic.

The overall prevalence of DR in treatment-experienced patients was 1.8% in Chengdu from 2014 to 2016, which was relatively lower than that in other areas of China, such as Jiangsu, similar to that in Yunnan and also lower than the average rate of Sichuan [31–33]. The DR rate in Chengdu for the past three years was 2.09%, 2.00%, and 1.50%, respectively, indicating that the ART treatment in Chengdu is relatively efficient.

CD4^+^ T cell count, CRF01 AE subtypes, and treatment with “TDF+3TC+EFV/NVP/other” are associated with DR. No significant differences were seen in other characteristics. Previous studies have suggested that patients with initial CD4^+^ T cell count > 200 cells/*μ*l had better recovery of immune function after ART [34, 35]. The level of CD4^+^ T cell count may reflect the function of the immune system, and the patients with poor immune function, that is, primary CD4^+^ T cell count ≤ 200 cells/*μ*l, are more likely to develop DR, indicating that HIV-infected patients should be detected early and initiate antiviral treatment started as soon as possible.

The Chengdu ART regimen includes 3TC, one NRTI (AZT/D4T/TDF), and one NNRTI (EFV/NVP). The resistance to NRTIs was DDI (69.80%), 3TC (69.39%), FTC (69.39%), ABC (69.39%), D4T (50.20%), TDF (47.76%), and AZT (13.47%) based on the number of the cases. Although FTC and DDI were not used in clinical settings, significant cross-resistance was observed. The structure, mechanism, and efficacy of FTC are similar to 3TC, and FTC was predicted to have the same DR profiles with 3TC. Similar findings were seen for DDI with ABC. Under the 3TC-based medication regimen, a high proportion of drug resistance was observed for 3TC, most of which showed high and intermediate resistance. Besides, the number of cases resistant to AZT was the lowest, most of which showed low or potential resistance. Thus, 3TC+AZT is the best choice in the current NRTI regimen.

In our study, M184I/V (59.59%) was the most prevalent mutation associated with NRTI resistance in our study and was also frequently found in Europe, Africa, and other regions in China [36, 37]. It alone causes high resistance to 3TC and FTC, low resistance to ABC, and potential resistance to DDI. K65R is one of the mutations with broad-spectrum resistance and was found in more than one-quarter of the cases. Nine mutations associated with DR were observed in large proportions and resistant to many NNRTIs. K101 E/H/P, Y181 C/V, and G190 A/E/K/Q/S/V are broad spectrum general mutations resistant to all NNRTIs. The L100I and M230L mutations (4.08%) had low incidence and intermediate or high resistance to RPV and ETR but no resistance to the first-line drugs EFV and NVP. These two mutations were found in CRF_01AE, CRF_07BC, and subtype C, and patients with these two mutations were undergoing 3TC+TDF+EFV/NVP, indicating that this regimen may be related to these mutations. Three primary mutations M46I, I47A, and I50V resistant to PIs were detected in one patient, whose regimen was 3TC+AZT+NVP then switched to LPV/r+3TC+TDF. This patient showed decreased treatment adherence that resulted in reduced effectiveness of treatment, indicating that clinicians should pay attention to compliance education to avoid similar cases and the transmission of “super” resistant strains.

Patients infected with the CRF01_AE are associated with DR. All 14 NRTI-associated mutations and 16 NNRTI-associated mutations were all found in CRF01_AE. Both cases identified with CRF55_01B showed DR, suggesting that this newly discovered CRF may likely develop DRMs. NRTI-associated DRMs M184I/V and K65R and NNRTI-associated DRMs with extensively drug resistance K101E/H/P, V179I/D/E/T, Y181C/V, and G190A/E/K/Q/S/V were detected in CRF55_01B. PI-associated mutations were found in 97 cases, most of which were secondary mutations like L10I/V, A71I/T/V, and K20I/R, so, there were relatively few cases of DR. In this study, among the PI-associated DR mutations detected, K20I/R and T74S were mainly found in CRF01_AE while A71I/T/V, Q58E, and V82I were frequently observed in CRF07_BC, indicating that the presence of different mutations may vary among different subtypes.

There are some limitations in this study. Part of the cases did not obtain available genotypes, and hence, sensitive methods may need to be explored to improve genotyping success rate. We observed that several factors are associated with HIVDR and then large sample size will be required to ensure the accuracy of this conclusion. In order to better apprehend and evaluate the prevalence of acquired HIVDR in this area, attention should be devoted to the prevalence of drug resistance in ART-naïve HIV-1 infected patients.

## 5. Conclusion

In this study, the HIV-1 genetic diversity and frequency of DRMs varies among individuals with ART failure in Chengdu. The overall prevalence of DR remained low in the studied population. Surveillance of VL and DR in patients receiving ART is of great significance, which can adjust treatment regimens and improve the quality of life. It also plays a considerable role to develop a clinical strategy for the prevention of the transmission of drug-resistant strains of HIV in this area.

## Figures and Tables

**Table 1:** Demographic characteristics and HIV-1 subtypes of the study participants.

	Participants	Subtypes					
		CRF01_AE	CRF07_BC	CRF08_BC	B	C	CRF55_01B
Total	481	261	200	9	6	3	2
Gender							
Male	411 (85.4)	222 (85.1)	175 (87.5)	4 (44.4)	5 (83.3)	3 (100.0)	2 (100.0)
Female	70 (14.6)	39 (14.9)	25 (12.5)	5 (55.6)	1 (16.7)	0 (0.0)	0 (0.0)
Age							
15~25	53 (11.0)	22 (8.2)	28 (14.0)	1 (11.1)	2 (33.3)	0 (0.0)	0 (0.0)
26~40	179 (37.2)	94 (36.0)	75 (37.5)	5 (55.6)	2 (33.3)	1 (33.3)	2 (100.0)
>40	249 (51.8)	145 (55.6)	97 (48.5)	3 (33.3)	2 (33.3)	2 (66.7)	0 (0.0)
Marital status							
Married/cohabiting	242 (50.3)	138 (52.9)	94 (38.8)	5 (55.6)	3 (50.0)	2 (66.7)	0 (0.0)
Unmarried	148 (30.8)	79 (30.3)	63 (42.6)	1 (11.1)	2 (33.3)	1 (33.3)	2 (100.0)
Divorced/widowed/separated	85 (17.7)	40 (15.3)	41 (48.2)	3 (33.3)	1 (16.7)	0 (0.0)	0 (0.0)
Unknown	6 (1.2)	4 (1.5)	2 (33.3)	0 (0.0)	0 (0.0)	0 (0.0)	0 (0.0)
Infection routes							
Heterosexual contact	342 (71.1)	189 (72.4)	138 (69.0)	9 (100.0)	4 (66.7)	2 (66.7)	0 (0.0)
Homosexual contact	98 (20.4)	49 (18.8)	45 (22.5)	0 (0.0)	2 (33.3)	0 (0.0)	2 (100.0)
Blood transfusion	1 (0.2)	0 (0.0)	1 (0.5)	0 (0.0)	0 (0.0)	0 (0.0)	0 (0.0)
Intravenous drug injection	7 (1.6)	3 (1.1)	4 (2.0)	0 (0.0)	0 (0.0)	0 (0.0)	0 (0.0)
Unknown	33 (6.9)	20 (7.7)	12 (6.0)	0 (0.0)	0 (0.0)	1 (33.3)	0 (0.0)

**Table 2:** Demographic characteristics of treatment-experienced HIV-1 individuals with virologic failure and univariate analyses for correlates of drug resistance.

Variables	Without DR	DR	*p* value
	*n* = 236	*n* = 245	
Gender			
Male	202 (85.6)	209 (85.3)	0.94
Female	34 (14.4)	36 (14.7)	
Age (years)			
15~	24 (10.2)	21 (8.6)	0.84
25~	63 (26.7)	67 (27.3)	
35~	47 (19.9)	57 (23.3)	
45~	33 (14.0)	36 (14.7)	
≥55	69 (29.2)	64 (26.1)	
Marital status			
Married/cohabiting	114 (48.3)	128 (52.2)	0.70
Single	74 (31.4)	74 (30.2)	
Divorced/widowed/separated	44 (18.6)	41 (16.7)	
Unknown	4 (1.7)	2 (0.8)	
Infection routes			
Heterosexual contact	166 (70.3)	176 (71.8)	0.83
Homosexual contact	51 (21.6)	47 (19.2)	
IDU	4 (1.7)	3 (1.2)	
Unknown	15 (6.4)	19 (7.8)	
CD4^+^ T cell count (cells/*μ*l)			
≤200	78 (33.1)	177 (72.2)	0.00
>200	151 (64.0)	65 (26.5)	
Unknown	7 (3.0)	3 (1.2)	
Treatment duration (year)			
0.5~	57 (24.2)	75 (30.6)	0.28
1~3	136 (57.6)	128 (52.2)	
≥3	43 (18.2)	42 (17.1)	
Treatment regimen			
AZT+3TC+EFV/NVP/others	91 (38.6)	64 (26.1)	0.00
D4T+3TC+EFV/NVP/others	26 (11.0)	24 (9.8)	
TDF+3TC+EFV/NVP/others	116 (49.2)	156 (63.7)	
3TC+EFV+NVP	3 (1.3)	0 (0.0)	
Unknown	0 (0.00)	1 (0.4)	
Treatment change			
Yes	8 (3.4)	17 (6.9)	0.07
No	228 (96.6)	228 (93.1)	
Viral load (log10)			
3~	130 (55.1)	111 (45.3)	0.10
4~	77 (32.6)	98 (40.0)	
≥3	29 (12.3)	36 (14.7)	
Subtypes			
CRF01_AE	94 (39.8)	167 (68.2)	0.00
CRF07_BC	131 (55.5)	69 (28.1)	
CRF08_BC	8 (3.4)	1 (0.4)	
CRF55_01B	0 (0.0)	2 (0.8)	
B	2 (0.8)	4 (1.6)	
C	1 (0.4)	2 (0.8)	

## Data Availability

All data generated or analyzed during this study are included in this article. All data and materials are presented in methods and results sections as shown in figures and tables. The data sets generated and/or analyzed during the current study are not publicly available due to policy of this project.

## Abbreviations

AIDS: Acquired immunodeficiency syndrome
HIV: Human immunodeficiency virus
WHO: World Health Organization
CRF: Circulating recombinant forms
VL: Virus load
NRTIs: Nucleoside reverse transcriptase inhibitors
NNRTIs: Nonnucleoside reverse transcriptase inhibitors
PIs: Protease inhibitors
3TC: Lamivudine
ABC: Abacavir
AZT: Zidovudine
D4T: Stavudine
TDF: Tenofovir
DDI: Didanosine
FTC: Emtricitabine
EFV: Efavirenz
NVP: Nevirapine
ETR: Etravirine
RPV: Rilpivirine
LPV/r: Lopinavir
ATV/r: Atazanavir
DRV/r: Darunavir
FPV/r: Fosamprenavir
IDV/r: Indinavir
NFV: Nelfinavir
SQV/r: Saquinavir
TPV/r: Tipranavir.

## Supplementary Materials

Supplementary Materials S Table 1: prevalence of DRMs in HIV-1 individuals with virologic failure in Chengdu from 2014 to 2016. S Table 2: distribution of HIV-1 DRMs in CRF01_AE and CRF07_BC.
