# Supplementary material for: HIV-1 Drug Resistance, Distribution of Subtypes, and Drug Resistance-Associated Mutations in Virologic Failure Individuals in Chengdu, Southwest China, 2014-2016
Source: Biomed Res Int. 2020 Mar 23;2020:5894124. doi: 10.1155/2020/5894124 (PMC7128060; doi:10.1155/2020/5894124)
Supplement: Supplementary Materials — S Table 1: prevalence of DRMs in HIV-1 individuals with virologic failure in Chengdu from 2014 to 2016. S Table 2: distribution of HIV-1 DRMs in CRF01_AE and CRF07_BC. [file 5894124.f1.pdf]

Supplementary material

S Table 1. Prevalence of DRMs in HIV-1 individuals with virologic failure in Chengdu from 2014 to 2016

Mutations	Prevalence of DR (n=245)					HIV-1 drug resistance level							
	2014	2015	2016	Total	%								
**PIs**						**LPV/r**	**DRV/r**	**FPV/r**	**IDV/r**	**NFV**	**SQV/r**	**TPV/r**	**ATV/r**
M46I	1	0	0	1	0.41								
I47A	1	0	0	1	0.41	H	I	H	L	H	L	I	L
I50V	1	0	0	1	0.41								
L10I/V	4	14	14	32	13.06	S	S	S	S	S	S	S	S
A71I/T/V	5	20	3	28	11.43	S	S	S	S	S	S	S	S
K20I/R	1	1	24	26	10.61	S	S	S	S	P	S	S	S
L33F/S	1	2	1	4	1.63	S	S	P/S	S	S	S	P/S	S
T74S	1	2	1	4	1.63	S	S	S	S	L	S	S	S
Q58E	1	1	1	3	1.22	S	S	S	S	S	S	L	S
V82I	0	0	2	2	0.82	S	S	S	S	S	S	S	S
**NRTIs**						**3TC**	**ABC**	**AZT**	**D4T**	**TDF**	**DDI**	**FTC**	
M184I/V	22	74	50	146	59.59	H	L	S	S	S	P	H	
K65R	9	40	20	69	28.16	I/H	I/H	S	I/H	I/H	I/H	I/H	
D67N/G	11	24	12	47	19.18	S	I/H	S	L/I	S	L/I	S	
K70E/K/R	8	19	15	42	17.14	L/I	L/I	S/L	L/I	L/I	L/I	L/I	
Y115F	5	18	14	37	15.10	S	H	S	S	S/P	S	S	
L74I/V	4	14	9	27	11.02	S	H	S	S	S	H	S	
T215I/Y	5	9	6	20	8.16	S	I/H	I/H	I/H	S	I/H	S	

V75I/L/M	5	10	4	19	7.76	S	S	S	S	S	P	S
A62V	1	6	7	14	5.71	S	S	S	S	S	S	S
K219E/Q	2	7	4	13	5.31	S	S	P	P	S	S	S
M41L	3	5	4	12	4.90	S	S	S/L	S/L	S	S/L	S
T69N/D	3	7	1	11	4.49	S	S	S	S	S	P	S
L210W	2	3	4	9	3.67	S	S	S	S	L/I	S	S
E44D	0	0	2	2	0.82	S	S	S	S	S	S	S
**NNRTIs**						**EFV**	**NVP**	**RPV**	**ETR**			
K103N	12	43	37	92	37.55	H	H	S	S/P			
G190A/E/K/Q/S/V	10	35	25	70	28.57	L/H	L/H	L/H	L/H			
V179I/D/E/T	10	32	26	68	27.76	P	P	P	P/L			
V106A/I/M	7	31	26	64	26.12	I/H	I/H	S	S			
Y181C/V	11	23	12	46	18.78	I/H	I/H	I/H	I/H			
K101E/H/P	1	22	13	36	14.69	L/H	L/H	L/H	L/H			
F227L	5	9	5	19	7.76	S	S	S	S			
V90I	5	13	0	18	7.35	S	S	S	S			
V108I	2	5	9	16	6.53	S	S	S	S			
E138A/G/R/K/Q	3	7	5	15	6.12	S	S	L	P			
H221Y	1	6	7	14	5.71	S	S	S	S			
Y188C/H/L	3	6	5	14	5.71	H	H	H	P/L			
L100I	0	7	3	10	4.08	S	S	I/H	I/H			
M230L	2	6	2	10	4.08	S	S	I/H	I/H			
P225H	2	3	5	10	4.08	S	S	S	S			
A98G	2	5	1	8	3.27	S	S	L	P			

NRTIs: Nucleoside reverse transcriptase inhibitors, NNRTIs: Non-nucleoside reverse transcriptase inhibitors, PIs: Protease Inhibitors,
S: susceptible, P: potential low-level resistance, L: low-level resistance, I: intermediate resistance, H: high-level resistance,
3TC: Lamivudine, ABC: Abacavir, AZT: Zidovudine, D4T: Stavudine, TDF: Tenofovir, DDI : Didanosine, FTC: Emtricitabine, EFV: Efavirenz, NVP: Nevirapine, ETR: Etravirine, RPV: Rilpivirine, LPV/r: Lopinavir, ATV/r: Atazanavir, DRV/r: Darunavir, FPV/r: Fosamprenavir, IDV/r: Indinavir, NFV: Nelfinavir, SQV/r: Saquinavir, TPV/r: Tipranavir

S Table2. Distribution of HIV-1 DRMs in CRF01_AE and CRF07_BC

Mutations	CRF01_AE		CRF07_BC	
	Cases	%	Cases	%
**NRTIs**				
M184I/V	107	43.67	33	13.47
K65R	45	18.37	20	8.16
D67N/G	40	16.33	6	2.45
K70E/K/R	33	13.47	8	3.27
Y115F	28	11.43	9	3.67
L74I/V	20	8.16	6	2.45
T215I/Y	16	6.53	3	1.22
V75I/L/M	17	6.94	0	-
A62V	11	4.49	3	1.22
K219E/Q	10	4.08	2	0.82
M41L	8	3.27	1	0.41
T69N/D	11	4.49	0	-
L210W	8	3.27	0	-
E44D	2	0.82	0	-
**NNRTIs**				
K103N	60	24.49	32	13.06
G190A/E/K/Q/S/V	52	21.22	15	6.12
V179I/D/E/T	50	20.41	14	5.71
V106A/I/M	44	17.96	18	7.35
Y181C/V	35	14.29	9	3.67
K101E/H/P	25	10.20	10	4.08
F227L	9	3.67	9	3.67
V90I	13	5.31	5	2.04
V108I	13	5.31	3	1.22
E138A/G/R/K/Q	12	4.90	1	0.41
H221Y	11	4.49	2	0.82
Y188C/H/L	10	4.08	4	1.63
L100I	7	2.86	3	1.22
M230L	7	2.86	2	0.82
P225H	5	2.04	5	2.04
A98G	8	3.27	0	-
**PIs**				
M46I	0	-	1	0.41
I47A	0	-	1	0.41
I50V	0	-	1	0.41
L10I/V	13	5.31	19	7.76
A71I/T/V	1	0.41	27	11.02
K20I/R	25	10.20	1	0.41

L33F/S	3	1.22	1	0.41
T74S	4	1.63	0	-
Q58E	0	-	3	1.22
V82I	0	-	2	0.82

NRTIs: Nucleoside reverse transcriptase inhibitors, NNRTIs: Non-nucleoside reverse transcriptase inhibitors, PIs: Protease Inhibitors
